# Plantar Spitz nevus mimicking melanoma^⋆^

**DOI:** 10.1016/j.abd.2020.10.024

**Published:** 2022-06-09

**Authors:** Guilherme Camargo Julio Valinoto, Felipe Henrique Yazawa Santos, Rute Facchini Lellis, Marcus Maia

**Affiliations:** a Dermatology Clinic, Santa Casa of São Paulo Hospital, São Paulo, SP, Brazil; b Laboratory of Pathology, Santa Casa of São Paulo Hospital, São Paulo, SP, Brazil

Dear Editor,

Spitz nevus is a benign melanocytic lesion with peculiar clinical, dermoscopic and histopathological features, which are often confused with those of melanoma, making its diagnosis a challenge. While melanocytic nevi are relatively common on the palmoplantar region, Spitz nevus rarely affects this site, with few reports in the literature.^1^

This case report describes a 20-year-old female patient, phototype III, with a complaint of a "spot" on the plantar surface for years, with recent growth. On the left plantar region, she had a blackened macula measuring 0.5 cm, with precise limits and irregular edges (Fig. 1). Dermoscopy showed a melanocytic lesion with a whitish blue veil in the center and peripheral brownish homogeneous areas, with a fibrillar pattern at one o'clock position (Fig. 2). An excisional biopsy was performed, with hypotheses of blue nevus and acrolentiginous melanoma. The anatomopathological examination revealed a compound fusocellular-epithelioid melanocytic nevus compatible with a Spitz nevus (Fig. 3).

**Figure 1:**
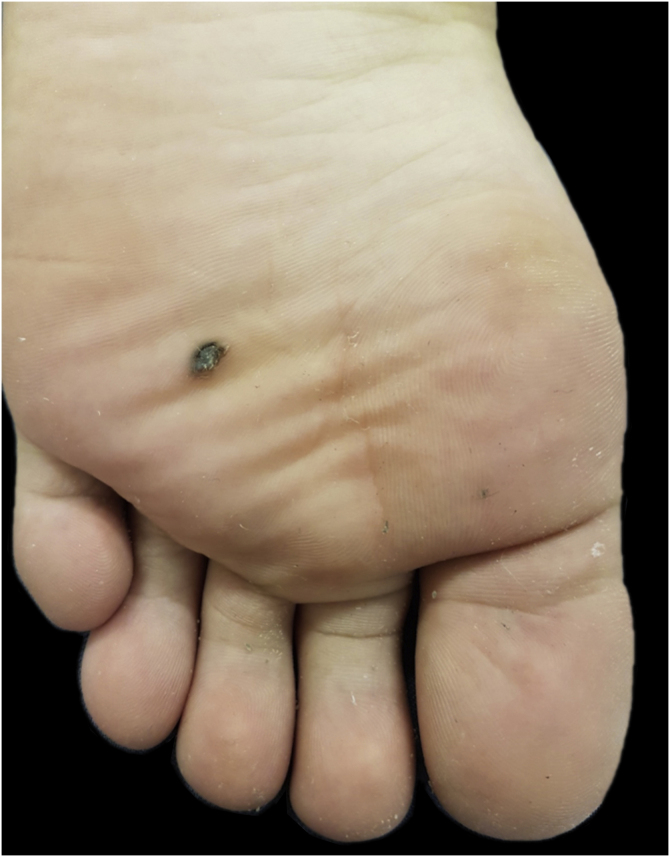
Blackened macule measuring 0.5 cm on the left plantar region.

**Figure 2:**
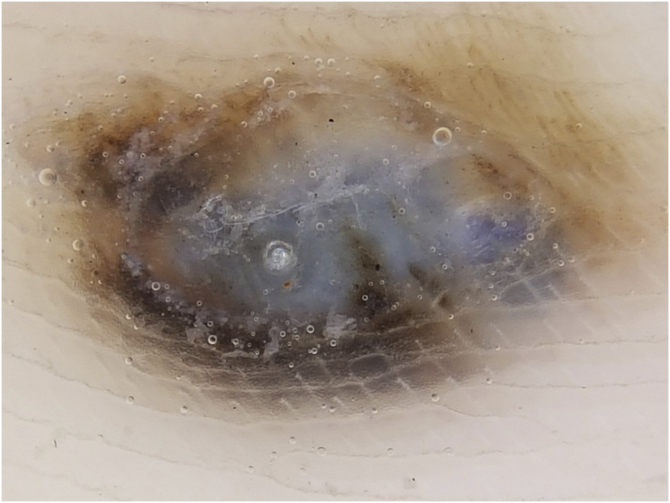
Dermoscopy showing a whitish-blue veil in the center and brownish pigment at the periphery, with a fibrillar pattern at one o'clock position.

**Figure 3:**
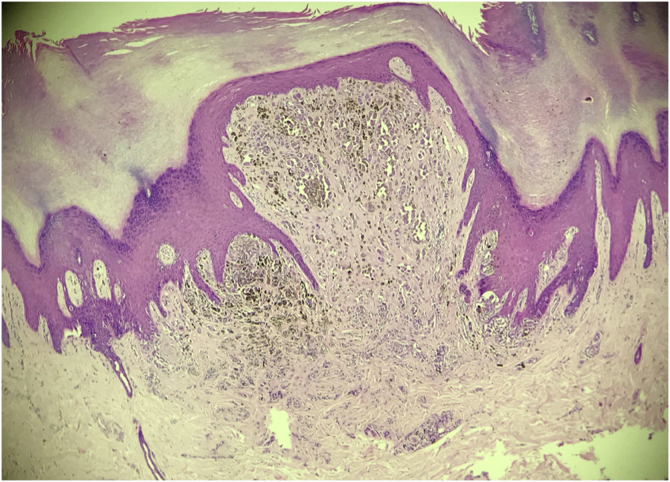
Histopathological examination evidencing a compound fusocellular-epithelioid melanocytic nevus with Spitz nevus characteristics (Hematoxylin & eosin, ×200).

According to a publication by Wiedemeyer et al. in 2018, acral Spitz nevus has a predilection for the plantar region of young female adults, a finding consistent with the present case. Moreover, it was observed that the acral variant is predominantly pigmented, with irregular borders and a larger size than conventional acral nevi. Such characteristics raise suspicion for malignancy, including atypical nevus and melanoma in the differential diagnosis.^1^

Dermoscopy is a valuable tool in the clinical diagnosis of pigmented lesions. Basically, in the case of acral melanocytic lesions, the parallel ridge pattern or diffuse, irregular pigmentation is highly suggestive of melanoma; the parallel furrow pattern prevails in benign melanocytic nevi.^2^, ^3^ Regarding acral Spitz nevus, however, it is possible to find varied patterns with more than one component, and the lack of specific findings makes it difficult to exclude malignancy.^4^

In parallel, the confocal reflectance microscopy (CRM) is a non-invasive imaging test that has helped to differentiate between benign nevi and melanomas. However, its use is limited in the investigation of acral lesions due to the palmoplantar stratum corneum thickness, which makes observation of deeper structures difficult.^5^

Histopathologically, the classic Spitz nevus presents large, spindle-shaped, and/or epithelioid melanocytes, with abundant eosinophilic cytoplasm, vesicular nucleus and small nucleolus. In acral Spitz nevus, which can be junctional or compound, a combination of spitzoid cytomorphology with the atypical junctional growth pattern of acral nevi is observed. It is also possible to find junctional nests with irregular distribution and shapes, pagetoid spread, and transepidermal elimination of melanocytic nests, findings that mimic the histopathological pattern of acrolentiginous melanoma.^4^ In dubious cases, the correct diagnosis can be aided by immunohistochemistry: Spitz nevi, with rare exceptions, express large amounts of P16 and P21 markers; conversely, acrolentiginous melanomas show significant loss of these markers.^1^

Therefore, while there are no studies that clarify the role of dermoscopy and CRM in differentiating between acral Spitz nevus and acrolentiginous melanoma, the excisional biopsy and histopathological study remain the diagnostic pillars for atypical acral pigmented lesions.

## Financial support

None declared.

## Authors' contributions

Guilherme Camargo Julio Valinoto: Drafting and editing of the manuscript; collection, analysis, and interpretation of data.

Felipe Henrique Yazawa Santos: Drafting and editing of the manuscript; collection, analysis, and interpretation of data.

Rute Facchini Lellis: Design and planning of the study; collection, analysis, and interpretation of data; effective participation in research orientation; critical review of the literature; critical review of the manuscript.

Marcus Maia: Approval of the final version of the manuscript; effective participation in research orientation; critical review of the literature; critical review of the manuscript.

## Conflicts of interest

None declared.

## References

[1] Wiedemeyer K., Guadagno A., Davey J., Brenn T. (2018). Acral Spitz Nevi: A Clinicopathologic Study of 50 Cases With Immunohistochemical Analysis of P16 and P21 Expression. Am J Surg Pathol..

[2] Saida T., Miyazaki A., Oguchi S., Ishihara Y., Yamazaki Y., Murase S. (2004). Significance of dermoscopic patterns in detecting malignant melanoma on acral volar skin: results of a multicenter study in Japan. Arch Dermatol..

[3] Kobayashi H., Oishi K., Miyake M., Nishijima C., Kawashima A., Kobayashi H. (2014). Spitz nevus on the sole of the foot presenting with transepidermal elimination. Dermatol Pract Concept..

[4] Vaccaro M., Borgia F., Cannavò S.P. (2015). Dermoscopy of pigmented variant of acral Spitz nevus. J Am Acad Dermatol..

[5] Cinotti E., Debarbieux S., Perrot J.L., Labeille B., Long-Mira E., Habougit C. (2016). Reflectance confocal microscopy features of acral lentiginous melanoma: a comparative study with acral nevi. J Eur Acad Dermatol Venereol..

